# Epidemiology, Population Health, and Health Impact Assessment

**DOI:** 10.2188/jea.JE20140212

**Published:** 2015-03-05

**Authors:** Gabriel Gulis, Yoshihisa Fujino

**Affiliations:** 1 University of Southern Denmark, Esbjerg, Denmark; 2 University of Occupational and Environmental Health, Kitakyushu, Fukuoka, Japan

**Keywords:** health impact assessment, social determinants of health, health inequity

The health of populations depends on many different factors. Epidemiology is a discipline that has a crucial role in describing health status, identifying risk factors, and analyzing relationships between health and different hazardous agents. The classical epidemiological triangle of host-agent-environment describes how individuals become ill. Disease occurs when an outside agent (vector) capable of causing disease or injury meets a host that is vulnerable to the agent. This happens in an environment that allows the agent and host to interact. Epidemiology not only measures the relationships between hosts and agents in certain environments but also analyses the health status of the population living in that environment.

As epidemiology is one of the essential disciplines of public health, its major aim is to contribute to fulfilment of the definition of public health as “a science and art to promote health and prevent disease by organized effort of society”.^1^ However, to improve the health status of the population, the knowledge produced by epidemiology needs to be used and translated into interventions. Epidemiology has a long tradition and has created a wealth of accumulated experience to assess micro-environments and specific agents that may impact health. However, epidemiology has been infrequently applied to assessment of public health issues at the policy or strategic level. In addition, epidemiology itself does not equip to deal with dialogue between stakeholders within its scientific discipline.

There are different types of interventions tackling all three elements of the triangle. One can work with hosts and improve their immune system, increase their knowledge, and motivate behavioral change to make the hosts more resistant to agents. Public health can also influence the presence and distribution of agents (vectors); this is often done via traditional hygiene measures, such as provision of safe drinking water, clean air, and good waste management, but also via anti-smoking regulations, diet advice, and physical activity guidelines. However, tackling the environment is a bit more difficult. If we consider the “micro-environment”, we are still on the host level and employ interventions like those mentioned above. If we are considering the “macro-environment”, as described, for example, by Dahlgren & Whitehead’s^2^ model of health (later modified by Barton and Grant^3^), different intervention methods need to be applied. This model of health is influenced by general political, social, and environmental conditions, and a set of social determinants of health, including work, education, culture, social cohesion, and individual behavior, as well as biological factors like age, sex, and genetics. Health impact assessment (HIA) aims to influence general social, political, and environmental factors, as well as the social determinants of health.

Because the goal of HIA is to assess potential future impacts of projects, plans, strategies, and policies on health,^4^ HIA projects intervene in the environment. Although there are several definitions of HIA and a confusion remains about what is and what is not HIA, the following three conditions must be met to be considered HIA:

1. A policy, project, programme, or plan is assessed and a decision upon it is expected to be taken;
2. Distribution of effects across the population is described; and
3. Dialogue between relevant stakeholders (stakeholders’ participation) is established.

HIA is based on values of democracy, equity, sustainable development, and ethical use of evidence.^4^ It is a multidisciplinary method that is open to experts of different disciplines (including epidemiologists, who are some of the key experts involved) and the public. All of these stakeholders constitute the steering group, which usually directs an HIA. These key conditions suggest how epidemiology contributes to HIA and why HIA is needed beyond epidemiology.

One should understand the environment in its broadest possible sense; the social, economic, cultural, political, and physical environments are equally included and equally relevant. HIA is a broad methodology, including both qualitative and quantitative methods, such as risk communication, risk assessment, and stakeholder analysis. Usually, HIA utilizes knowledge gathered by basic disciplines of public health, such as epidemiology, to outline potential health impacts and quantify them. However, HIA can work in the opposite way as well; HIA often identifies areas where we know little about the interaction of hosts and agents in a specific environment. In other words, HIA cannot be done without substantial contributions from epidemiologists but can help to identify concrete relationships where we lack quantitative knowledge and provide epidemiology with new research themes. Epidemiology is the sole contributor to the second point of the three key aspects of HIA; it provides evidence-based knowledge on the distribution of health effects and their risk factors across different population groups. On the other hand, without dialogue among stakeholders, epidemiology may overlook the fact that decision-making is based on not only scientific evidence, but also on political, economic, and social considerations; HIA adds this element to the value of epidemiology. HIA, through its key values (democracy, equity, sustainable development, and ethical use of evidence) and direct link to decision- and policy-making processes, is also considered an effective mechanism for implementing the precautionary principle.^5^

Many examples are available in published literature to illustrate the link between epidemiology and HIA. One of most profound is the cement kiln case from Rugby municipality evaluated by Cook and Kemm in 2002 (http://www.apho.org.uk/resource/item.aspx?RID=44206). In that case, an industrial company decided to modify their technology, which raised concerns in the municipality and led to a request to conduct an HIA of the proposal. The HIA, using epidemiological evidence from studies on air pollution and health, identified and assessed the key pollutant and suggested an overall impact. The assessment satisfied the needs of the company and also addressed the concerns of the municipality.

There is some discussion regarding the similarities and differences between HIA and other methods or disciplines in public health. The difference between HIA and epidemiology is clear concerning the three points described above; however, it is necessary to understand how HIA differs from evaluation, risk assessment, and other impact assessments, such as environmental impact assessment. HIA does not evaluate actions; it assesses potential future impacts of new policies, plans, and programs, which is a clear difference between HIA and evaluation. Risk assessment is usually a standardized procedure mostly focusing on a single chemical or other toxic agent; HIA usually deals with a mixture of different substances, environments, and determinants, including social determinants. HIA uses knowledge and information from individual risk assessment studies and balances them in a risk appraisal step. Compared to environmental impact assessment, which focuses on elements of the environment, HIA focuses on health effects and impacts caused by different determinants (again including social determinants).

As with any discipline, HIA requires well-trained experts. Being a relatively new discipline, training for HIA is not yet as broadly recognized as that for epidemiology. However, regular training courses are organized by universities around the world (eg, University of Liverpool, Liverpool, United Kingdom; University of New South Wales, Sydney, Australia; University of Southern Denmark, Esbjerg, Denmark; and University of Occupational and Environmental Health, Fukuoka, Japan) and information can be found at the “HIA gateway” (http://www.apho.org.uk/default.aspx?QN=P_HIA). The World Health Organization is actively supporting HIA through information distribution, organization of national training workshops on demand of its member states, and publication activity.^6^ Literature on HIA, both in terms of methodology and experience from around the world, has grown substantially over the last three years.^7^^–^^9^

In conclusion, a close collaboration of epidemiologists and health impact assessment experts is a “must”. Such collaboration can support the development of public health and have a long-term positive impact on population health.
